# Supplementary material for: Peptaibol Production and Characterization from Trichoderma asperellum and Their Action as Biofungicide
Source: J Fungi (Basel). 2022 Sep 29;8(10):1037. doi: 10.3390/jof8101037 (PMC9605287; doi:10.3390/jof8101037)
Supplement: Supplementary file 1 [file jof-08-01037-s001.zip › jof-1874794-supplementary.pdf]

Journal of Fungi

Supplementary Materials

Table S1. Absolute and relative abundance of the most common amino acids in the sequence of P*aib* produced by *Trichoderma* species

Aminoacid	Absolute abundance	Relative abundance (%)
Aib	1156	36.9
Leucine/Isoleucine	465	14.8
Glutamine	319	10.2
Valine/Isovaline	305	9.7
Alanine	282	9.0
Proline	261	8.3
Glycine	145	4.6
Phenylalanine	72	2.3
Serine	43	1.4
Asparagine	38	1.2
Glutamate	32	1.0
Tryptophan	10	0.3
Others	8	0.3
Total	3136	100

Source: based in the information reported at the P_{aib} Database [55]

Table S2. Regression coefficients and probabilities associated with the factors in the model for predicting the production of P_{aib}.

Factor	Coeficient	Probability
Constant	4.05E+08	<0.001*
Aib (A)	4.28E+08	0.0002*
F. oxysporum (F)	-4.51E+08	0.7637
Interaction (A-F)	-2.85E+07	0.4842
Aib quadratic (A-A)	-5.78E+07	0.0887
F. oxysporum quadratic (F-F)	1.45E+08	0.0016*

*Indicates the variable has a significative effect over the observed response (P < 0.05).
